# No Association Between Suicidality and Weight Among School-Attending Adolescents in the United Arab Emirates

**DOI:** 10.3389/fpsyg.2021.618678

**Published:** 2021-03-17

**Authors:** Hania Ibrahim, Ziyad R. Mahfoud

**Affiliations:** ^1^Department of Medical Education, Weill Cornell Medicine–Qatar, Doha, Qatar; ^2^Division of Epidemiology, Department of Population Health Sciences, Weill Cornell Medicine, New York City, NY, United States

**Keywords:** suicide, adolescents, obesity, weight, suicide ideation, suicide attempts

## Abstract

Previous data on the link between weight and suicidality is heterogenous. We aim to investigate the potential association between weight and suicidality among adolescents in the United Arab Emirates (UAE). We hypothesize that an association exists between weight and suicidality, with those at both extremes of weight suffering higher rates of suicidal ideation, planning and attempts. The 2016 UAE Global School Health Survey (GSHS) was used. Weight categories based on the World Health Organization Body Mass Index charts were generated. Suicidality measures were based on questions on suicide ideation, planning, and attempt. Univariate and multivariate binary logistic regression were used. Overall, 3.6, 21.4, and 17.5% of students were found to be underweight, overweight, and obese, respectively. In total, 492 students (14.6%) reported suicidal ideation, 397 (11.8%) reported planning, and 389 (11.4%) reported attempts within the twelve months prior to the survey. In the multivariate logistic regression, being female, older, and of lower socioeconomic status were significantly associated with increased suicidal ideation, planning and attempts. Increased parental involvement was associated with decreased suicidality. The association between weight category and suicidality did not reach statistical significance. A significant proportion of Emirati teens are under- or over-weight, with one in seven and one in nine having considered or attempted suicide, respectively. However, this study finds no significant association between weight and suicide ideation, planning, or attempts. This may be due to cultural differences in weight perception. Further research into this association can aid in tailoring suicide prevention interventions.

## Introduction

Adolescent and childhood obesity are rising worldwide (James, 2004). During the last three decades, there has been a concerning rise in the number of overweight and obese children and teenagers (Han et al., 2010). Among Western countries, this trend has been well-studied. In the United States upward of 30% of children and teens are overweight or at risk of becoming overweight (James, 2004). The rise in adolescent weight is linked to a steady rise in caloric intake combined with lower rates of physical activity (Goran and Treuth, 2001). On the other hand, being underweight is also common, especially among adolescents. This poses its own set of health risks that may lead to medical complications.

Obesity in general, including adolescent and childhood obesity, has also become a widespread phenomenon in Gulf countries. In the United Arab Emirates (UAE), the 14.7% of teens are overweight, another 18.9% are obese while 7.6% of school-age children are underweight (Al Junaibi et al., 2013). In total, existing data suggests that upward of 40% of school children in the UAE are on the extremes of weight.

The physical health complications of both obesity and low weight are well-studied. Overweight adolescents experience higher risk of fatty liver disease, obstructive sleep apnea, diabetes mellitus and high blood pressure. These are chronic complications of obesity that have a long-term impact on quality of life, morbidity, and mortality (Kumar and Kelly, 2017). For those on the opposite end of the spectrum, being underweight is associated with stunted growth, which can continue into adulthood (Moradi et al., 2019).

However, the mental health complications associated with extremes of weight are less established. A previous international study established an association between overweight and depression (Quek et al., 2017). Another study established a direction of this association, finding that depression led to obesity more often than obesity led to depression (Mannan et al., 2016). One Dutch study determined that elevated BMI was linked to anxiety, depression, attention problems, and problematic behavior among adolescents (Ter Bogt et al., 2006). Previous literature suggests that those who complete or attempt suicide are characterized by a number of risk factors experienced by those who are overweight, which include mood disorders, interpersonal conflict, limited social support, and hopelessness (Serafini et al., 2012). Although the pathophysiology between mood disorders and weight has not been fully established, one possible explanation to consider is the role of sensory processing in predicting poor outcomes among those with major depressive disorders (Serafini et al., 2017).

Despite the existence of some literature on mental health and weight, data on weight and suicide in adolescents remains limited in the Gulf and globally. While a large-scale meta-analysis among adults found that obesity had an inverse association with suicide mortality and attempts (Amiri and Behnezhad, 2018), a Swedish study found that 15-year-old obese boys were three times more likely to attempt suicide as peers of normal weight (Berg et al., 2005).

The link between being underweight and suicide has not been well-established. As food accessibility and agricultural practices continue to improve in the Middle East, causes of low weight shift from food scarcity to congenital or mental health causes: namely growth delays or eating disorders. While a prominent mood component is often present in adolescents with eating disorders, little data is generated on underweight teens beyond those with anorexia nervosa.

The main aim of the study is to determine if there is an association between weight category and suicidality among school-going adolescents in the UAE. We hypothesize that abnormal weight categories will be linked to higher rates of suicidal ideation, planning and attempts, with the highest suicidality found among the obese and overweight groups.

## Materials and Methods

### Study Sample

The UAE is classified as a high-income country and is located in the Persian Gulf. The country’s population is approximately 9,990,000. The UAE’s official language is Arabic, and immigrants make up the majority of the population at 87.9% (Central Intelligence Agency, 2020). The UAE has a large proportion of children and adolescents, with a youth dependency ratio of 17.7. The literacy rate is 93.8% (Central Intelligence Agency, 2020).

This is a secondary data analysis of the open-access de-identified data from the Global School Health Survey (GSHS), a cross-sectional survey conducted by the Center for Disease Control (CDC) and the World Health Organization (WHO). The survey was administered to a representative sample of students enrolled in both private and public secondary schools across the UAE, with most students between the ages of 13 to 17 years. The CDC does not report exact data collection milestones, but data was collected within the calendar year of 2016. Survey sampling utilized a two-stage cluster sampling design from a representative proportion of schools within the UAE, including all Emirates, public, and private schools. The first stage of sampling involved selecting schools with probability proportional to the size of enrollment while the second stage involved randomly selected classrooms in which all students were sampled (Ministry of Health, United Arab Emirates, 2016). The GSHS placed an emphasis on the following outcomes of adolescent physical and mental health: diet, physical activity, mental health, hygiene, violence, sexual behavior, and substance abuse. The questionnaire was composed of 59 self-administered questions covering various adolescent health outcomes. Inclusion criteria included being enrolled in secondary school between grades 7–12, speaking English or Arabic, and agreeing to fill out the survey. The only exclusion criteria was declining to complete the survey. A total of 5,849 adolescents completed the 2016 GSHS survey for the UAE, for a school response rate of 94% (Ministry of Health, United Arab Emirates, 2016). However, data on suicidality was only available for about 3400 of them.

### Weight Categories

Typically, the survey administrators would determine the measurements of each student before starting the survey, which would then be written for the student on a slip of paper (Centers for Disease Control and Prevention, 2014). The GSHS questionnaire asked participants for self-reported height and weight using the initial measurements as a guide, which were then used to calculate body mass index. This method of self-reporting was found to be highly reliable and valid, providing a reasonable proxy for the determination of participants’ weight status (Brener et al., 2003). Body Mass Index (BMI) was calculated using the reported height and weight, and data for each student was compared to WHO BMI charting of the same age and gender to determine weight category (World Health Organization, 2007).

### Suicidal Ideation, Planning, and Attempts

Suicidality was measured using self-reported survey questions. Questions about seriously considering suicide, planning for suicide, and the number of attempts were asked. For ideation and planning, responses were collected as a yes/no answer. For attempts, the survey gathered the number of previous attempts; this question was then dichotomized into a yes/no outcome, with 1 or more attempts considered yes.

The following questions were used in the analysis:

• During the past 12 months, did you ever seriously consider attempting suicide?
• During the past 12 months, did you make a plan about how you would attempt suicide?
• During the past 12 months, how many times did you actually attempt suicide?

### Demographic Variables

Demographic variables that were available for use included age, gender, grade level, and school type (public vs. private), food security and variables related to the adolescent’s relationship with his/her parents. Food security was based on the question “During the past 30 days, how often did you go hungry because there was not enough food in your home?” with possible answers of “never,” “rarely,” “sometimes,” “most of the time,” or “always”. Adolescent-parent relationship was assessed using the following four questions:

• During the past 30 days, how often did your parents or guardians check to see if your homework was done?
• During the past 30 days, how often did your parents or guardians understand your problems and worries?
• During the past 30 days, how often did your parents or guardians really know what you were doing with your free time?
• During the past 30 days, how often did your parents or guardians go through your things without your approval?

All questions have responses of “never,” “rarely,” “sometimes,” “most of the time,” or “always” that were dichotomized to yes/no with a cut-off of “most of the time” and Always “always” denoting adequate parental involvement (= yes), except for question 4, where “never” and “rarely” were used to determine healthy parental boundaries.

### Statistical Analysis

Demographic variables related to the adolescent population sampled were summarized using frequency distributions. The distribution of weight categories was computed and compared within the levels of each of the demographic variables using Chi-squared tests. Associations between the three suicidality variables (ideation, planning, and attempt) and weight categories were done using binary logistic regressions. Multiple logistic regressions were performed adjusting for all available demographics of the participant: age, gender, grade, school type, food security, and relationship with the parents after making sure that there were no collinearity issues that might affect model fitting. Both unadjusted and adjusted odds ratios along with their 95% confidence intervals and *p*-values were reported. Listwise missing value deletion was used during the analysis. Data analysis was conducted using IBM SPSS (version 26, Armonk, New York, United States). Significance was set at the 5% level.

## Results

### Sample Characteristics

The vast majority of the participants were between the ages of 13 and 17 (88.4%) from grades 8 to 12 (95.7%). The majority of the participants were females (52.4%), from private schools (50.6%), and reported never being hungry at some point in the last month due to a lack of food at home (51.2%) (Table 1).

**TABLE 1:** Participant demographics and association with weight category.

	Total	Underweight	Normal	Overweight	Obese
	*N* (%)	*N* (%)	*N* (%)	*N* (%)	*N* (%)
	5826 (100.0)	192 (3.6)	3074 (57.4)	1148 (21.4)	939 (17.5)
Gender (*p* < 0.001)*					
Male	2763 (47.6)	124 (4.9)	1337 (52.7)	518 (20.4)	556 (21.9)
Female	3041 (52.4)	68 (2.4)	1737 (61.6)	630 (22.4)	383 (13.6)
Age (*p* = 0.001)*					
11 to 12	322 (5.5)	2 (0.7)	132 (48.7)	79 (29.2)	58 (21.4)
13	911 (15.6)	33 (4.0)	454 (55.3)	195 (23.8)	139 (16.9)
14	1126 (19.3)	42 (4.1)	589 (57.8)	220 (21.6)	168 (16.5)
15	1153 (19.8)	37 (3.5)	611 (57.5)	233 (21.9)	182 (17.1)
16	1089 (18.7)	31 (3.0)	604 (58.7)	221 (21.5)	173 (16.8)
17	871 (15.0)	29 (3.5)	492 (60.0)	151 (18.4)	148 (18.0)
18	354 (6.1)	18 (5.5)	192 (58.2)	49 (14.8)	71 (21.5)
Grade Level (*p* = 0.038)*					
7	244 (4.3)	7 (3.2)	104 (47.9)	67 (30.9)	39 (18.0)
8	1215 (21.2)	45 (4.1)	639 (58.7)	223 (20.5)	182 (16.7)
9	1156 (20.2)	39 (3.7)	578 (55.0)	242 (23.0)	191 (18.2)
10	1207 (21.1)	38 (3.4)	628 (57.0)	237 (21.5)	199 (18.1)
11	976 (17.0)	28 (3.0)	541 (58.4)	206 (22.2)	151 (16.3)
12	935 (16.3)	32 (3.6)	527 (60.1)	157 (17.9)	161 (18.4)
School Type (*p* < 0.001)*					
Private	2962 (50.6)	91 (3.4)	1565 (57.8)	629 (23.2)	423 (15.6)
Public	2887 (49.4)	101 (3.8)	1509 (57.1)	519 (19.6)	516 (19.5)
**Food Security (*p* = 0.123)**					
Never	2979 (51.2)	101 (3.7)	1597 (57.9)	595 (21.6)	465 (16.9)
Rarely	1172 (20.1)	26 (2.4)	630 (59.2)	230 (21.6)	179 (16.8)
Sometimes	1085 (18.7)	45 (4.5)	534 (53.9)	217 (21.9)	194 (19.6)
Most of the time or Always	581 (10.0)	20 (3.8)	302 (58.1)	102 (19.6)	96 (18.5)
Parents checked homework (*p* = 0.006)*					
Yes	2489 (43.7)	92 (4.0)	1292 (56.2)	470 (20.5)	444 (19.3)
No	3209 (56.3)	98 (3.3)	1718 (58.5)	649 (22.1)	470 (16.0)
**Parents understood problems (*p* = 0.739)**					
Yes	2610 (46.1)	88 (3.6)	1404 (58.1)	518 (21.4)	406 (16.8)
No	3052 (53.9)	99 (3.6)	1589 (57.1)	596 (21.4)	500 (18.0)
**Parents knew about free time (*p* = 0.817)**					
Yes	3010 (52.9)	106 (3.8)	1587 (57.2)	598 (21.6)	482 (17.4)
No	2676 (47.1)	83 (3.4)	1421 (58.0)	518 (21.1)	429 (17.5)
**Parent never/rarely went through things (*p* = 0.257)**					
Yes	4474 (78.9)	138 (3.3)	2391 (57.8)	889 (21.5)	718 (17.40)
No	1193 (21.1)	49 (4.6)	603 (56.3)	230 (21.5)	190 (17.7)

*Significant at the 5% level.

Overall, 38.9% (95%CI: 34.6–37.1%) of students were overweight or obese while only 3.6% (95%CI: 2.8–3.8%) were underweight.

### Association Between Weight Category and Participants’ Demographics

There were significant associations between weight category and gender, age, grade, and type of school but not with the food security. In particular, males were significantly more likely to be obese or underweight as compared to females. The prevalence of overweight was higher in grades 7 (67.0%), 9 (23.0%), 10 (21.5%), and 11 (22.2%) as compared to grade 12 (17.9%; *p*-value = 0.038). The prevalence of obesity was significantly higher in the 18-years-olds (21.5%) as compared to the 14-year-olds (16.5%; *p*-value ≤0.001).

Obesity was significantly higher in the public schools (19.5%) as compared to the private ones (15.6%; *p*-value ≤0.001) (see Table 1). One element of parental involvement was associated with weight categories, specifically checking homework (*p*-value = 0.006), while other aspects of parental involvement such as knowing whereabouts (*p*-value = 0.817), understanding worries (*p*-value = 0.739) and privacy (*p*-value = 0.0.257) were not significantly associated with weight. Food security, a proxy to socioeconomic status, was not found to be associated with weight category (*p*-value = 0.123).

### Suicide-Related Outcomes

**FIGURE 1:**
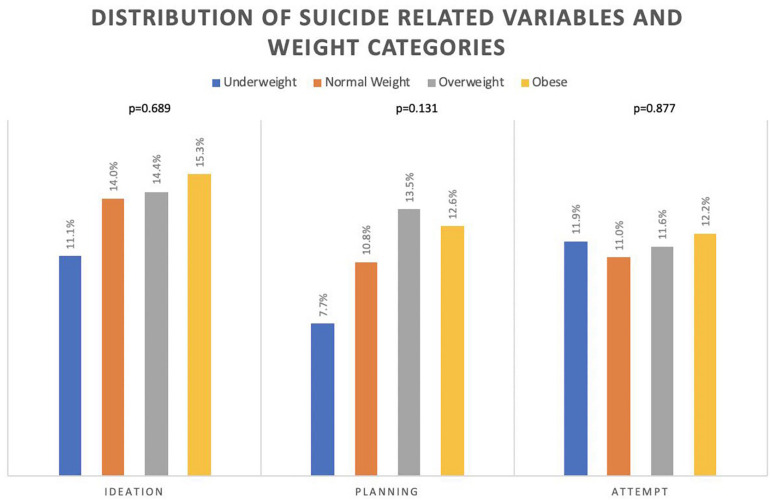
The distribution of suicide ideation, planning, and attempts among weight categories.

The overall prevalence of suicide ideation, planning and attempts were 14.6% (95%CI: 13.4–15.8%), 11.8% (95%CI: 10.7–12.9%) and 11.4% (95%CI: 10.3–12.5%), respectively. Although the prevalence of all suicide-related outcomes was the highest among the abnormal weight categories, especially the obese or the overweight weight categories, the associations between suicide ideation, planning, or attempts and weight category didn’t reach statistical significance (*p*-values = 0.689, 0.131, and 0.877, respectively; see Figure 1).

### Association Between Suicide-Related Outcomes and Participants’ Demographics and Weight Category: Bivariate and Multivariate Analysis

Since adolescents in the different weight categories had significant differences in demographics, we repeated the above comparisons using bivariate and multivariate logistic regressions, adjusting for those potential confounders.

Unadjusted and adjusted odds ratios along with their 95% confidence intervals (CI) and p-values are presented in Tables 2–4. The multivariate logistic regression models did not reveal a statistically significant association between weight category and suicide ideation or attempts. However, those who were overweight had significantly higher odds of suicide planning as compared to those with normal weight (AOR = 1.380, 95%CI: 1.035–1.840).

**TABLE 2:** Bivariate and multivariate results of the association between suicide ideation and weight category with other demographics of participants.

Suicide ideation									

		Unadjusted OR and 95% C.I. for OR				Adjusted OR and 95% C.I. for AOR			
	Yes-*N* (%)	OR	Lower	Upper	*p*-value	AOR	Lower	Upper	*p*-value
**Age**									
11 to 12 years old	9 (1.8)	0.385	0.180	0.822	0.014*	0.242	0.083	0.701	0.009*
13 years old	56 (11.5)	0.626	0.404	0.969	0.036*	0.433	0.207	0.910	0.027*
14 years old	94 (19.2)	0.806	0.539	1.206	0.294	0.609	0.315	1.180	0.142
15 years old	98 (20.0)	0.751	0.504	1.119	0.159	0.501	0.276	0.908	0.023*
16 years old	110 (22.5)	0.96	0.647	1.424	0.838	0.830	0.489	1.409	0.491
17 years old	81 (16.6)	0.806	0.534	1.217	0.305	0.797	0.501	1.267	0.336
18 years old or older	41 (8.4)	1.000				1.000			
**Gender**									
Male	199 (40.8)	1.216	1.000	1.477	0.049*	0.794	0.627	1.004	0.054
Female	289 (59.2)	1.000				1.000			
**Grade**									
Grade 7	1 (0.2)	0.364	0.048	2.77	0.329	0.685	0.082	5.716	0.727
Grade 8	112 (23.3)	1.092	0.803	1.486	0.574	2.481	1.314	4.685	0.005*
Grade 9	83 (17.3)	0.900	0.649	1.25	0.53	1.447	0.807	2.595	0.215
Grade 10	125 (26.0)	1.206	0.892	1.631	0.225	1.707	1.052	2.771	0.030*
Grade 11	77 (16.0)	1.039	0.743	1.454	0.821	1.087	0.707	1.672	0.704
Grade 12	82 (17.1)	1.000				1.000			
**Food security (Hunger)**									
Never	195 (40.0)	1.000				1.000			
Rarely	105 (21.5)	1.481	1.147	1.913	0.003*	1.256	0.942	1.675	0.121
Sometimes	105 (21.5)	1.709	1.321	2.212	<0.001*	1.453	1.091	1.934	0.011*
Most of the time or Always	83 (17.0)	2.741	2.051	3.664	<0.001*	2.112	1.520	2.934	<0.001*
**Weight**									
Normal weight	264 (58.8)	1.000				1.000			
Underweight	13 (2.9)	0.767	0.425	1.385	0.379	0.786	0.414	1.491	0.461
Overweight	91 (20.3)	1.03	0.797	1.333	0.820	1.078	0.816	1.423	0.599
Obese	81 (18.0)	1.105	0.843	1.447	0.470	1.191	0.888	1.597	0.243
**School**									
Private	199 (40.4)	0.630	0.519	0.765	<0.001*	0.769	0.608	0.973	0.029*
Public	293 (59.6)	1.000				1.000			
**Parental Involvement**									
Checked homework	158 (32.7)	1.980	1.615	2.428	<0.001*	0.762	0.600	0.968	0.026*
Understood problems	126 (25.9)	0.318	0.256	0.394	<0.001*	0.450	0.349	0.581	<0.001*
Knew about free time	181 (37.4)	2.381	1.952	2.905	<0.001*	0.611	0.482	0.774	<0.001*
Never or rarely went through their things	346 (71.6)	1.929	1.547	2.406	<0.001*	0.543	0.422	0.699	<0.001*

*Significant at the 5% level.

**TABLE 3:** Bivariate and multivariate results of the association between suicide planning and weight category with other demographics of participants.

Suicide Planning									

		Unadjusted OR and 95% C.I. for OR				Adjusted OR and 95% C.I. for AOR			
	Yes-*N* (%)	OR	Lower	Upper	*p*-value	AOR	Lower	Upper	*p*-value
**Age**									
11 to 12 years old	10 (2.5)	0.482	0.231	1.006	0.052	0.198	0.067	0.589	0.004*
13 years old	40 (10.1)	0.485	0.301	0.782	0.003*	0.241	0.109	0.533	<0.001*
14 years old	73 (18.5)	0.688	0.449	1.055	0.087	0.422	0.210	0.848	0.015*
15 years old	81 (20.5)	0.692	0.454	1.054	0.086	0.517	0.277	0.966	0.039*
16 years old	84 (21.3)	0.796	0.523	1.211	0.287	0.655	0.377	1.138	0.134
17 years old	70 (17.7)	0.777	0.505	1.197	0.253	0.709	0.442	1.139	0.155
18 years old or older	37 (9.4)	1.00				1.000			
**Gender**									
Male	154 (39.0)	0.768	0.620	0.952	0.016*	0.722	0.559	0.933	0.013*
Female	241 (61.0)	1.000				1.000			
**Grade**									
Grade 7	2 (0.5)	0.873	0.197	3.875	0.859	1.658	0.331	8.307	0.538
Grade 8	86 (22.2)	0.912	0.655	1.270	0.585	2.513	1.278	4.942	0.008*
Grade 9	73 (18.8)	0.882	0.625	1.245	0.476	1.657	0.896	3.062	0.107
Grade 10	89 (22.9)	0.923	0.664	1.282	0.631	1.189	0.704	2.007	0.517
Grade 11	64 (16.5)	0.943	0.660	1.348	0.749	0.971	0.618	1.525	0.898
Grade 12	74 (19.1)	1.000				1.000			
**Food security**									
Never	166 (41.9)	1.000			<0.001*	1.000			
Rarely	85 (21.5)	1.377	1.043	1.819	0.024*	1.320	0.973	1.79	0.074
Sometimes	76 (19.2)	1.389	1.041	1.855	0.026*	1.257	0.918	1.723	0.154
Most of the time or Always	69 (17.4)	2.547	1.868	3.472	<0.001*	1.799	1.26	2.568	0.001*
**Weight**									
Normal Weight	203 (55.8)	1.000				1.000			
Underweight	9 (2.5)	0.688	0.343	1.380	0.293	0.582	0.262	1.293	0.184
Overweight	85 (23.4)	1.286	0.981	1.686	0.069	1.380	1.035	1.840	0.028*
Obese	67 (18.4)	1.193	0.888	1.602	0.241	1.299	0.949	1.779	0.103
**School**									
Private	158 (39.8)	0.613	0.495	0.759	<0.001*	0.780	0.605	1.007	0.056
Public	239 (60.2)	1.000				1.000			
**Parental Involvement**									
Checked homework	147 (37.6)	0.652	0.525	0.811	<0.001*	0.929	0.721	1.198	0.570
Understood problems	126 (32.1)	2.205	1.763	2.759	<0.001*	0.629	0.482	0.821	0.001*
Knew about free time	160 (40.8)	0.504	0.407	0.624	<0.001*	0.615	0.476	0.795	<0.001*
Never or rarely went through their things	275 (70.5)	0.500	0.394	0.635	<0.001*	0.500	0.383	0.653	<0.001*

*Significant at the 5% level.

**TABLE 4:** Bivariate and multivariate results of the association between attempting suicide and weight category with other demographics of participants.

Suicide attempts									

		Unadjusted OR and 95% C.I. for OR				Adjusted OR and 95% C.I. for AOR			
	Yes-*N* (%)	OR	Lower	Upper	*p*-value	AOR	Lower	Upper	*p*-value
**Age**									
11 to 12 years old	6 (1.5)	0.263	0.108	0.641	0.003*	0.069	0.020	0.241	<0.001*
13 years old	42 (10.8)	0.474	0.297	0.756	0.002*	0.122	0.056	0.266	<0.001*
14 years old	82 (21.1)	0.727	0.48	1.100	0.132	0.256	0.129	0.507	<0.001*
15 years old	83 (21.3)	0.662	0.438	1.000	0.050	0.345	0.186	0.640	0.001*
16 years old	74 (19.0)	0.642	0.422	0.978	0.039*	0.441	0.252	0.770	0.004*
17 years old	63 (16.2)	0.643	0.417	0.991	0.045*	0.638	0.397	1.026	0.064
18 years old or older	39 (10.0)	1.000				1.000			
**Gender**									
Male	176 (45.7)	1.030	0.833	1.275	0.784	0.964	0.750	1.239	0.773
Female	209 (54.3)	1.000				1.000			
**Grade**									
Grade 7	4 (1.0)	2.290	0.732	7.162	0.154	4.268	1.001	18.191	0.050
Grade 8	94 (24.5)	1.140	0.815	1.593	0.444	5.929	3.041	11.562	<0.001*
Grade 9	79 (20.6)	1.082	0.765	1.532	0.655	3.197	1.733	5.897	<0.001*
Grade 10	86 (22.4)	1.003	0.714	1.410	0.985	1.956	1.153	3.32	0.013*
Grade 11	55 (14.3)	0.907	0.621	1.324	0.613	1.266	0.797	2.011	0.318
Grade 12	66 (17.2)	1.000				1.000			
**Food security**									
Never	165 (42.7)	1.000				1.000			
Rarely	81 (21.0)	1.304	0.984	1.729	0.065	1.130	0.830	1.536	0.438
Sometimes	80 (20.7)	1.474	1.109	1.959	0.008*	1.158	0.846	1.585	0.360
Most of the time or Always	60 (15.5)	2.138	1.549	2.95	<0.001*	1.494	1.042	2.143	0.029*
**Weight**									
Normal Weight	210 (57.7)	1.000				1.000			
Underweight	14 (3.8)	1.085	0.610	1.931	0.781	0.935	0.494	1.770	0.837
Overweight	74 (20.3)	1.058	0.798	1.402	0.696	1.102	0.815	1.489	0.527
Obese	66 (18.1)	1.125	0.838	1.511	0.434	1.132	0.826	1.550	0.441
**School**									
Private	138 (35.5)	0.506	0.407	0.631	<0.001*	0.558	0.431	0.722	<0.001*
Public	251 (64.5)	1.000				1.000			
**Parental Involvement**									
Checked homework	150 (39.6)	0.725	0.583	0.902	0.004*	0.991	0.770	1.277	0.945
Understood problems	115 (30.1)	0.416	0.331	0.524	<0.001*	0.581	0.444	0.760	<0.001*
Knew about free time	149 (39.1)	0.475	0.382	0.591	<0.001*	0.620	0.480	0.802	<0.001*
Never or rarely went through their things	277 (73.1)	0.598	0.468	0.765	<0.001*	0.638	0.484	0.841	0.001*

*Significant at the 5% level.

On the other hand, age was consistently associated with the three suicide outcomes, with older age showing higher risk of suicidality. In particular, those in age groups 11–13, and 15 had significantly lower odds of suicide ideation as compared to those who are 18 years old (AOR = 0.242 with 95%CI: 0.083–0.701; AOR = 0.433 with 95%CI:0.207–0.901; and AOR = 0.501 with 95%CI:0.276–0.908, respectively). Similar trends were observed for suicide planning (AOR = 0.198, with 95%CI:0.067–0.589 for 11–12 years old) and attempts (AOR = 0.069 with 95%CI:0.020–0.241 for 11–12 years old). Along with age, grade level was associated with ideation, planning and attempts. Specifically, Grade 8 students presented with a consistently higher risk for suicidality (for ideation, AOR = 2.481, 95%CI: 1.314–4.685; planning AOR = 2.513, 95%CI:1.278–4.942; attempts AOR = 5.929, 95%CI: 3.041–11.562).

Compared to females, male adolescents reported lower rates of suicide planning (AOR = 0.722, 95%CI: 0.559–0.993). No significant gender differences were observed for suicide ideation or attempts. Low food security was associated with a significant increase in ideation, planning, and attempts (for ideation, AOR = 2.112, 95%CI: 1.520–2.934, for planning AOR = 1.799, 95%CI:1.260–2.568, and attempts AOR = 1.494, 95%CI: 1.042–2.143).

Compared to adolescents in public schools, those in private schools reported significantly lower likelihood of suicide ideation and attempts (for ideation, AOR = 0.769, 95%CI: 0.608–0.973 and for attempts AOR = 0.558, 95%CI:0.431–0.722). A similar trend was observed for suicide planning, but statistical significance was not reached (AOR = 0.780, 95%CI:0.605–1.007).

Finally, positive forms of parental involvement; such as understanding problems, knowing whereabouts, and not going through personal belongings were significantly associated with less suicide ideation, planning and attempts (see Tables 2–4). For example, adolescents who reported that their parents understood their problems, reported a significant 42% reduction in the odds of suicide attempts (AOR = 0.581, 95%CI: 0.444–0.760).

## Discussion

The prevalence of being underweight (3.6%), overweight (21.4%), and obese (17.5%) found in this study suggests that a large proportion of the UAE’s adolescent population falls in an abnormal weight category. This data on weight distribution is comparable to previous estimates of obesity in the country (Al-Haddad et al., 2000). Moreover, rates of obesity were comparable to other countries of the Gulf Region, such as Kuwait (23.1%) and Bahrain (17.9%) (Ministry of Health, Bahrain, 2016; Ministry of Health, Kuwait, 2016).

In terms of suicidality, the overall prevalence of suicide ideation in the UAE (14.6%) was close to previous data for the UAE from 2005 (12.7%) (Mahfoud et al., 2011). The prevalence of suicide ideation was also comparable to other countries in the region including Lebanon (16.0%), Jordan (16.6%) (Mahfoud et al., 2011). While limited data exist on the reasons for high suicide ideation despite previous reports of low rates of completed suicides among the UAE and other countries, several factors have been considered to be linked to such a discrepancy. One such factor is the role of the predominant religious belief system, Islam, which strongly prohibits suicide. Another factor is close family connections, as reflected in our findings of the value of parental involvement in serving as a protective factor (Mahfoud et al., 2011). While data is limited on the protective nature of parental involvement, data from previous literature supports this finding (Mahfoud et al., 2011).

Our study did not find a significant association between weight categories and suicide ideation or attempts among adolescents in the UAE. Except for the association between planning and being overweight; no other weight categories showed a statistically significant link with suicidality. Our findings are consistent with a previous American study on high school students that indicated that BMI was not associated with suicide ideation, but that weight perception had a significant impact on such ideation (Eaton et al., 2005). The results of a Korean study supported these findings and reported a weak connection between BMI and suicidal ideation when controlling for body weight perception, thereby establishing the latter as a potential mediator (Kim et al., 2009). These findings may shine a light on the cultural connotations of weight in the Gulf and specifically in the UAE, and how these weight perceptions can influence mental health broadly and suicidality specifically. One previous study found that close to half of overweight adolescents in the UAE perceived themselves to be of average weight. Furthermore, nearly one third of non-overweight or non-obese teens felt pressured by their parents to gain weight (Musaiger et al., 2012). These findings, coupled with the lack of association between weight and suicide ideation, point to a difference in perception of weight and culture surrounding weight in this country.

Paradoxically, cultural norms dictate that higher weight is connected to wealth and health in Emirati culture. One study found that overweight students of a Middle Eastern background were less likely to label themselves as “too fat” as compared to their Caucasian or Asian counterparts (O’Dea, 2008). This is supported by studies that show that 63.5% of Emirati parents with overweight children misclassified their children’s weight group, indicating that parental perception is as inaccurate as adolescents’ (Aljunaibi et al., 2013). These findings may suggest that the relationship between weight and poor mental health is dictated by the cultural, parental, and personal perception of weight and not by the absolute weight itself.

These findings also point to a number of other findings that are congruent with previous literature. Suicide ideation rises with age, as adolescents develop a sense of identity and personality and face greater challenges. The rise in suicidal ideation with age is consistent with results from the Quebec Longitudinal Study, which reports an increase in suicidal ideation throughout adolescence (Orri et al., 2020). Late adolescents face more scrutiny and responsibility, and this time marks a time of heightened risk for new-onset major depressive disorder and other comorbid mental health conditions that may worsen suicidality (Carpenter et al., 2000). Moreover, food security is closely linked to socioeconomic status, a well-established risk factor for suicide ideation. Female students reported higher rates of suicide planning, a finding congruent with previous studies that suggest that females’ risk of depression and suicide ideation increases significantly in adolescence and continues into adulthood (Mannan et al., 2016). While our study did not unearth direct causes, one explanation for the discrepancy between suicidality in males versus females are the prominent gender roles within the region which one study hypothesized may have an adverse effect on mental health outcomes among women (Al Junaibi et al., 2013).

This study has several limitations. For one, the use of a cross-sectional database limits the ability to establish causality between the selected variables. Moreover, while BMI was pre-recorded, it was self-reported thereby limiting accuracy. Suicidal ideation, planning, and attempts were also self-reported and serious attempts were not compared to previous medical history records. This may lead to over- or under-reporting of such attempts; however, the study found a similar in rate of suicidality as compared to other Arab countries (Mahfoud et al., 2011). The GSHS database also lacked questions on potential confounding mental health conditions, such as eating disorders or substance abuse, which could affect both weight and suicide ideation to attempts. Finally, the large proportion of missing responses to the questions on suicide could make the results of this study not generalizable to all school attending adolescents in UAE. That said, compared to those who answered the suicide-related questions, those with missing responses were more likely to be males, younger, in lower grades, and overweight or obese.

In conclusion, this study found that there was no clear link between weight and suicidality among adolescents in the UAE, while multiple other factors were associated with higher reports of suicidality including: female gender, older age, and lower socioeconomic status. Weight perception is a possible explanation for the lack of link between weight and suicidality in the UAE as compared to previous literature in other regions. The results of the study can help guide recommendations for mental health practitioners in the UAE on which factors contribute most to suicide ideation, planning and attempts. Future public health research and interventions aimed at reducing suicide ideation or attempts should explore the possible role of socioeconomic status, older age, gender, and poor parental involvement on suicide variables. These factors may be used as stratifiers in the primary care setting to help in determining vulnerability and thereby provide more targeted care to adolescents.

## Data Availability Statement

Publicly available datasets were analyzed in this study. This data can be found here: https://extranet.who.int/ncdsmicrodata/index.php/catalog/71/get_microdata.

## Author Contributions

ZM contributed to the conceptualization of the study and guided the data analysis and write up of the manuscript. HI contributed to the data analysis and write up of the manuscript. Both authors reviewed and approved the final version of the manuscript.

## Conflict of Interest

The authors declare that the research was conducted in the absence of any commercial or financial relationships that could be construed as a potential conflict of interest.

## References

[B1] Al Junaibi A. Abdulle A. Sabri S. Hag-Ali M. Nagelkerke N. (2013). The prevalence and potential determinants of obesity among school children and adolescents in Abu Dhabi, United Arab Emirates. *Int. J. Obes.* 37 68–74. 10.1038/ijo.2012.131 22890490

[B2] Aljunaibi A. Abdulle A. Nagelkerke N. (2013). Parental weight perceptions: a cause for concern in the prevention and management of childhood obesity in the United Arab Emirates. *PLoS One* 8:e59923. 10.1371/journal.pone.0059923 23555833 PMC3608558

[B3] Al-Haddad F. Al-Nuaimi Y. Little B. B. Thabit M. (2000). Prevalence of obesity among school children in the United Arab Emirates. *Am. J. Hum. Biol.* 12 498–502. 10.1002/1520-6300(200007/08)12:4<498::AID-AJHB9>3.0.CO;2-P 11534041

[B4] Amiri S. Behnezhad S. (2018). Body mass index and risk of suicide: a systematic review and meta-analysis. *J. Affect. Disord.* 238 615–625. 10.1016/j.jad.2018.05.028 29957479

[B5] Berg I. M. Simonsson B. Ringqvist I. (2005). Social background, aspects of lifestyle, body image, relations, school situation, and somatic and psychological symptoms in obese and overweight 15-year-old boys in a county in Sweden. *Scand. J. Prim. Health Care* 23 95–101. 10.1080/02813430510015313 16036548

[B6] Brener N. D. McManus T. Galuska D. A. Lowry R. Wechsler H. (2003). Reliability and validity of self-reported height and weight among high school students. *J. Adolesc. Health* 32 281–287. 10.1016/S1054-139X(02)00708-5 12667732

[B7] Carpenter K. M. Hasin D. S. Allison D. B. Faith M. S. (2000). Relationships between obesity and DSM-IV major depressive disorder, suicide ideation, and suicide attempts: results from a general population study. *Am. J. Public Health* 90 251–257. 10.2105/AJPH.90.2.251 10667187 PMC1446144

[B8] Centers for Disease Control and Prevention (2014). *Global School-Based Student Health Survey: 2013 GSHS Data User’s Guide.* Atlanta, GA: Centers for Disease Control and Prevention.

[B9] Central Intelligence Agency. (2020). *United Arab Emirates. In The World Factbook.* Langley, VA: Central Intelligence Agency

[B10] Eaton D. K. Lowry R. Brener N. D. Galuska D. A. Crosby A. E. (2005). Associations of body mass index and perceived weight with suicide ideation and suicide attempts among US high school students. *Arch. Pediatr. Adolesc. Med.* 159 513–519. 10.1001/archpedi.159.6.513 15939848

[B11] Goran M. I. Treuth M. S. (2001). Energy expenditure, physical activity, and obesity in children. *Pediatr. Clin. North Am.* 48 931–953. 10.1016/S0031-3955(05)70349-7 11494644

[B12] Han J. C. Lawlor D. A. Kimm S. Y. (2010). Childhood obesity. *Lancet* 375 1737–1748. 10.1016/S0140-6736(10)60171-7 20451244 PMC3073855

[B13] James P. T. (2004). Obesity: the worldwide epidemic. *Clin. Dermatol.* 22 276–280. 10.1016/j.clindermatol.2004.01.010 15475226

[B14] Kim D. S. Cho Y. Cho S. I. Lim I. S. (2009). Body weight perception, unhealthy weight control behaviors, and suicidal ideation among Korean adolescents. *J. Sch. Health* 79 585–592. 10.1111/j.1746-1561.2009.00452.x 19909422

[B15] Kumar S. Kelly A. S. (2017). Review of childhood obesity: from epidemiology, etiology, and comorbidities to clinical assessment and treatment. *Mayo Clin. Proc.* 92 251–265. 10.1016/j.mayocp.2016.09.017 28065514

[B16] Mahfoud Z. R. Afifi R. A. Haddad P. H. DeJong J. (2011). Prevalence and determinants of suicide ideation among Lebanese adolescents: results of the GSHS Lebanon 2005. *J. Adolesc.* 34 379–384. 20434762 10.1016/j.adolescence.2010.03.009

[B17] Mannan M. Mamun A. Doi S. Clavarino A. (2016). Prospective associations between depression and obesity for adolescent males and females-a systematic review and meta-analysis of longitudinal studies. *PLoS One* 11:e0157240. 10.1371/journal.pone.0157240 27285386 PMC4902254

[B18] Ministry of Health, Bahrain (2016). *Global School-based Student Health Survey Bahrain 2016 Fact Sheet.* Available online at: https://cdn.who.int/media/docs/default-source/ncds/ncd-surveillance/data-reporting/bahrain/gshs/gshs-fs-bahrain-government-2016.pdf?sfvrsn=42ff9e67_2&download=true

[B19] Ministry of Health, Kuwait (2016). *Global School-based Student Health Survey Kuwait 2016 Fact Sheet*. Available online at: https://www.who.int/ncds/surveillance/gshs/2015_GSHS_Kuwait_Fact_Sheet.pdf

[B20] Ministry of Health, United Arab Emirates (2016). *Global School-based Student Health Survey UAE 2016 Fact Sheet*. Available online at: https://www.who.int/ncds/surveillance/gshs/UAE-2016-gshs-fact-sheet.pdf?ua=1

[B21] Moradi S. Mirzababaei A. Mohammadi H. Moosavian S. P. Arab A. Jannat B. (2019). Food insecurity and the risk of undernutrition complications among children and adolescents: a systematic review and meta-analysis. *Nutrition* 62 52–60. 10.1016/j.nut.2018.11.029 30852458

[B22] Musaiger A. O. Zaal A. B. D’souza R. (2012). Body weight perception among adolescents in Dubai, United Arab Emirates. *Nutr. Hosp.* 27 1966–1972. 23588446 10.3305/nh.2012.27.6.5830

[B23] O’Dea J. A. (2008). Gender, ethnicity, culture and social class influences on childhood obesity among Australian schoolchildren: implications for treatment, prevention and community education. *Health Soc. Care Commun.* 16 282–290. 10.1111/j.1365-2524.2008.00768.x 18328051

[B24] Orri M. Scardera S. Perret L. C. Bolanis D. Temcheff C. Séguin J. R. (2020). Mental health problems and risk of suicidal ideation and attempts in adolescents. *Pediatrics* 146:e20193823. 10.1542/peds.2019-3823 32513840

[B25] Quek Y. H. Tam W. W. Zhang M. W. Ho R. C. (2017). Exploring the association between childhood and adolescent obesity and depression: a meta−analysis. *Obes. Rev.* 18 742–754. 10.1111/obr.12535 28401646

[B26] Serafini G. Gonda X. Canepa G. Pompili M. Rihmer Z. Amore M. (2017). Extreme sensory processing patterns show a complex association with depression, and impulsivity, alexithymia, and hopelessness. *J. Affect. Disord.* 210 249–257. 10.1016/j.jad.2016.12.019 28064114

[B27] Serafini G. Pompili M. Innamorati M. Rihmer Z. Sher L. Girardi P. (2012). Can cannabis increase the suicide risk in psychosis? A critical review. *Curr. Pharm. Des.* 18 5165–5187. 10.2174/138161212802884663 22716157

[B28] Ter Bogt T. F. van Dorsselaer S. A. Monshouwer K. Verdurmen J. E. Engels R. C. Vollebergh W. A. (2006). Body mass index and body weight perception as risk factors for internalizing and externalizing problem behavior among adolescents. *J. Adolesc. Health* 39 27–34. 10.1016/j.jadohealth.2005.09.007 16781958

[B29] World Health Organization (2007). *Growth Reference Data for 5–19 Years. 2007.* Geneva: World Health Organization.

